# Does native *Trypanosoma cruzi* calreticulin mediate growth inhibition of a mammary tumor during infection?

**DOI:** 10.1186/s12885-016-2764-5

**Published:** 2016-09-13

**Authors:** Paula Abello-Cáceres, Javier Pizarro-Bauerle, Carlos Rosas, Ismael Maldonado, Lorena Aguilar-Guzmán, Carlos González, Galia Ramírez, Jorge Ferreira, Arturo Ferreira

**Affiliations:** 1 Institute of Biomedical Sciences (ICBM), Faculty of Medicine, University of Chile, Avenida Independencia 1027, Independencia, Santiago, Chile; 2 Faculty of Veterinary Medicine and Livestock Sciences, University of Chile, Avenida Santa Rosa 11735, La Pintana, Santiago, Chile; 3 Faculty of Veterinary Medicine, Andrés Bello University, Avenida República 440, Santiago Centro, Santiago, Chile; 4 University of Chile, Avenida Independencia 1027, Santiago, Chile

**Keywords:** Mammary tumor, *Trypanosoma cruzi*, Calreticulin, Angiogenesis

## Abstract

**Background:**

For several decades now an antagonism between *Trypanosoma cruzi* infection and tumor development has been detected. The molecular basis of this phenomenon remained basically unknown until our proposal that *T. cruzi* Calreticulin (TcCRT), an endoplasmic reticulum-resident chaperone, translocated-externalized by the parasite, may mediate at least an important part of this effect. Thus, recombinant TcCRT (rTcCRT) has important in vivo antiangiogenic and antitumor activities. However, the relevant question whether the in vivo antitumor effect of *T. cruzi* infection is indeed mediated by the native chaperone (nTcCRT), remains open. Herein, by using specific modified anti-rTcCRT antibodies (Abs), we have neutralized the antitumor activity of *T. cruzi* infection and extracts thereof, thus identifying nTcCRT as a valid mediator of this effect.

**Methods:**

Polyclonal anti-rTcCRT F(ab’)_2_ Ab fragments were used to reverse the capacity of rTcCRT to inhibit EAhy926 endothelial cell (EC) proliferation, as detected by BrdU uptake. Using these F(ab’)_2_ fragments, we also challenged the capacity of nTcCRT, during *T. cruzi* infection, to inhibit the growth of an aggressive mammary adenocarcinoma cell line (TA3-MTXR) in mice. Moreover, we determined the capacity of anti-rTcCRT Abs to reverse the antitumor effect of an epimastigote extract (EE). Finally, the effects of these treatments on tumor histology were evaluated.

**Results:**

The rTcCRT capacity to inhibit ECs proliferation was reversed by anti-rTcCRT F(ab’)_2_ Ab fragments, thus defining them as valid probes to interfere in vivo with this important TcCRT function. Consequently, during infection, these Ab fragments also reversed the in vivo experimental mammary tumor growth. Moreover, anti-rTcCRT Abs also neutralized the antitumor effect of an EE, again identifying the chaperone protein as an important mediator of this anti mammary tumor effect. Finally, as determined by conventional histological parameters, in infected animals and in those treated with EE, less invasive tumors were observed while, as expected, treatment with F(ab’)_2_ Ab fragments increased malignancy.

**Conclusion:**

We have identified translocated/externalized nTcCRT as responsible for at least an important part of the anti mammary tumor effect of the chaperone observed during experimental infections with *T. cruzi*.

**Electronic supplementary material:**

The online version of this article (doi:10.1186/s12885-016-2764-5) contains supplementary material, which is available to authorized users.

## Background

In this report, we identify a protein from the *Trypanosoma cruzi* (the protozoan agent of Chagas disease) endoplasmic reticulum (ER), as responsible for, at least an important part of the antitumor effect of this infection.

Chagas’ disease (American Trypanosomiasis) is mainly spread by *Triatominae* insects. Originally confined to America, the disease has now gone global [1].

Only 30 % of *T. cruzi* infected people presents variable symptoms, years or decades after infection [1], thus indicating that the protozoan components, as occurs in many parasitisms, are relatively well tolerated during the infection.

Interestingly, several reports indicate that in patients with Chagas’ disease cancer is an extremely rare event, in particular breast adenocarcinomas. Thus, about 80 years ago, Roskin, Ekzempliarskaia and Klyuyeva, researchers from the former Soviet Union, postulated an experimental anticancer toxic activity derived from this infection. When they inoculated *T. cruzi* extracts, directly in a peritumoral area, in different tumors, both in experimental animals and in humans, similar results to those obtained with the infection were generated. Moreover, the parasite capacity to infect preferentially tumor cells, as compared to normal host cells, was also described (reviewed in [2]). Although, in general, these data suggest an antagonism between *T. cruzi* infection and tumor growth [3], research progress in these areas was seriously hampered by the intense international political problems of those years (i.e. the Cold War) [4, 5]. Although several publications on these issues have appeared during the last decade [3, 6–8], the molecular basis of this phenomenon has remained elusive.

Our laboratory interest in understanding molecular terms ruling the host/parasite interplay has led us to the identification, sequencing, cloning, expression and characterization of a 45 kDa protein, *T. cruzi* Calreticulin (TcCRT), an ER-resident chaperone [9–14]. We have described three domains in recombinant TcCRT (rTcCRT): N-terminal (N-TcCRT) (aa 120–180), with antiangiogenic activity [9, 15]; central-S (aa 159–281), that binds and inhibits human C1, the first component of the complement system, [16] and, P (aa173–286), mainly involved in calcium binding [16].

Most important, during infection TcCRT is exteriorized from the ER to the area of flagellum emergence [16]. We have shown that rTcCRT inhibits angiogenesis (in vitro*, ex vivo, in ovum* and in vivo) in three vertebrate species (*Rattus rattus, Gallus gallus* and *Homo sapiens sapiens*) [9, 15, 17], and that it interferes with morphogenesis, migration and proliferation of endothelial cells (ECs) [9]. When ECs were incubated with rTcCRT, we observed that the protein was internalized. This internalization is inhibited by fluid-phase Fucoidan [9, 10], a sulfated polysaccharide and ligand for Scavenger Receptors (SR) [18].

The parasite cycle in the mammal host implies that, from the original infection site caused by the hematophagous arthropod vector, *T. cruzi* must access the circulation in order to reach its target tissues (mainly heart, esophagus, colon and aorta) [1]. Once in the circulation, the parasite must swiftly contact the ECs apical membrane surface. In this key step and since ECs display receptors (cC1qR) for complement component C1 [19, 20], a synapse will be formed by parasite TcCRT, host C1 and EC (host) CRT (cC1qR) (reviewed in [2]). Alternatively or concomitantly, *T. cruzi* ability to contact and infect ECs may involve direct interaction of TcCRT with a constitutive SR present on ECs [21–23].

rTcCRT, inoculated in a peritumoral area, reduced the growth of an aggressive, multiresistant mammary adenocarcinoma (TA3-MTXR) in mice [9]. The rTcCRT antiangiogenic activity and antitumor effects were more efficient than those mediated by human CRT (rHuCRT), when compared at equimolar concentrations [9, 17].

However, these facts do not necessarily mean that native parasite CRT (nTcCRT), indeed mediates the infection antineoplastic effect. This important question is justified by the following rationale: i). The possibility exists that one or several, still unidentified parasite molecules, could largely mediate the important antitumor effects observed during *T. cruzi* infection (or after injection of parasite lysates); ii). Given the cloning procedures, recombinant (rTcCRT) used in vivo in the previously reported experiments [14], is structurally different from the native version operating during infection (e.g.: addition of a polyhistidine tail, lack of glycosylations, marginal LPS contamination, protein degradation, among other possibilities); iii). The experimental conditions of the reported in vivo treatment of tumor-bearing animals with rTcCRT [9] (i.e. the protein is injected several times in peritumor sites) are radically different from those mediated by an experimental or natural infection with the parasite and, iv). CRTs from different species were traditionally located in the ERs, and all of them carry a carboxiterminal ER-retention signal (KDEL, in TcCRT) [19, 24]. Although nTcCRT is translocated to the parasite area of flagellum emergence [15], we have no firm vidence that the chaperone can diffuse to the surrounding fluid phase. Antibody (Ab) -targeted neutralization of its antitumor effects during infection is thus informative with regard to this important function.

Herein, we propose that, during *T. cruzi* infection, the responsible antitumor molecule is nTcCRT expressed by the parasite. We have been able to revert the antitumor effect of the infection by targeting nTcCRT with specific Abs, among the multitude of parasite molecules present in the infecting parasite (or extracts thereof).

## Methods

### Reagents, recombinant proteins and Abs

rTcCRT and its N-TcCRT domain, rabbit whole anti-rTcCRT Abs, their preimmune counterparts, as well as their F(ab’)_2_ fragments, were all generated, in our laboratory, by immunizing rabbits with the pure recombinant molecule, by standard procedures [9, 15, 25]. All these Abs were monospecific since, in immunowestern assays they recognized only nTcCRT in whole *T. cruzi* extracts. The rationale for choosing whole anti-rTcCRT Abs or their F(ab’)_2_ fragments, in the experiments described below, is examined in the Discussion section.

### Capacity of F(ab’)_2_ anti-rTcCRT Ab fragments to prevent the chaperone binding to human ECs

The experimental design aimed at studying the capacity of anti-rTcCRT F(ab’)_2_ Ab fragments to neutralize the interaction of the parasite chaperone with human ECs. Two types of Fucoidan were used as positive controls [9]. First, 2x10^5^ ECs (EAhy926) [26] (donated by Dr. Gareth Owen, Pontifical Catholic University of Chile), were seeded in IMDM (Iscove’s Modified Dulbecco’s Medium, Invitrogen, USA). The medium was supplemented with 10 % v/v heat-inactivated fetal bovine serum (FBS, Invitrogen, USA), 1 % penicillin/streptomycin (Sigma, USA), 1 % glutamine (Invitrogen, USA) and sodium bicarbonate 0.3 M (MERCK, Germany). The cells were collected at 80 % confluence and expanded. rTcCRT was coupled to FITC (F-rTcCRT) according to manufacturer’s instructions (FluoReporter FITC Protein Labeling Kit, Molecular Probes, USA).

Later, in eight in vitro experimental groups, 3×10^5^ EAhy926 cells were incubated in 96-well flat bottom plates, for 6 h, at 37 °C, adding: i.) F-rTcCRT (1 μM); ii.) F-rTcCRT with anti-rTcCRT F(ab’)_2_ fragments (120 μg); iii.) F-rTcCRT and preimmune Ab fragments; iv.) F-rTcCRT, with Fucoidan 1 (Mw = 20–200 kDa) (Sigma-Aldrich, USA) (100 μg); v.) F-rTcCRT, with Fucoidan 2 (Mw = 58.6 kDa) (Sigma-Aldrich, USA) (100 μg); vi.) N-rTcCRT; vii.) N-rTcCRT with Fucoidan 1 and, viii.) N-rTcCRT with Fucoidan 2.

After 6 h incubations, the cells were analyzed by flow cytometry (BD FACSAria III) (20.000 events) using the software FCS Express 5.

### Capacity of F(ab’)_2_ anti-rTcCRT Ab fragments to revert the rTcCRT-dependent inhibition of EC proliferation

The EAhy926 cells origin, culture and harvesting conditions were described above. Reversion of the anti-proliferative rTcCRT effect on ECs was detected by BrdU uptake (BrdU Cell Proliferation Assay Kit, Millipore© cat. 2750). Briefly, 3×10^4^ ECs were incubated in 96-well flat bottom plates (Nunc, UK), for 24, 48 and 72 h. ECs were the incubated with rTcCRT (1 μM) and/or 80 μg in 200 μl of F(ab’)_2_ fragments (anti-rTcCRT or preimmune controls). Results were expressed as percentage compared to the control group (100 % proliferation).

### Tumor, induction and evaluation

The methotrexate (MTX)-resistant (MTXR) cell line (TA3-MTXR) [27], was originally isolated from the TA3 mammary adenocarcinoma, grown as ascites by weekly i.p. injections of 10^6^ cells into histocompatible mice [28]. The TA3-MTXR mammary tumor cell line was generated by weekly consecutive selection in the presence of MTX [28]. The tumor cells TA3-MTXR used herein were extracted from mouse ascites, diluted in physiological saline solution and centrifuged at 423G for 5 min at room temperature. The supernatant was discarded and the pellet was suspended in physiological saline, and counted in a Neubauer chamber. Over 95 % tumor cell viability was routinely obtained.

In all experiments, mice were inoculated s.c. on the supra scapular region, with 5×10^5^ TA3-MTXR, freshly obtained mammary tumor cells, contained in 100 μl.

Evaluation of tumor growth was blindly monitored, for 30 days [11] and, in compliance with bioethical regulations, until they reached a maximum of 3.000 mm^3^, when the animals were euthanized. In each test, the minimum number of animals per experimental group was eight, unless otherwise defined, a calculation based on mean values and their standard deviations, obtained in previous experiments [29].

### Immune mediated specific reversion of the antitumor effects of rTcCRT

Five experimental groups were designed. All of them were inoculated with the TA3-MTXR tumor cells. In addition the animals were treated as follows: Group I: PBS, as positive control (s.c.); Group 2: rTcCRT (50 μg, s.c.); Group 3: In order to reverse the rTcCRT antitumor capacity, this group was inoculated with whole anti-rTcCRT Abs (500 μg); Group 4: As a negative control these mice received preimmune Abs and, Group 5: N-rTcCRT.

All inoculations were performed every other day on a peritumor area.

### Immune mediated specific reversion of the antitumor effects of a trypomastigote infection

Four experimental groups were designed. All of them were inoculated with the TA3-MTXR tumor cells. In addition the animals were treated as follows: Group I: PBS, as positive control (i.v); Group 2: Mice were infected i.p. with 10^3^ trypomastigotes (infective form) of the Tulahuén strain; Group 3: In order to reverse the antitumor capacity of the infection, mice were also inoculated, in the lateral tail vein, with F(ab’)_2_ anti-rTcCRT Abs (200 μg in 100 μl) and, Group 4: As a negative control, these mice received preimmune F(ab’)_2_ Abs. All groups were inoculated with TA3-MTXR tumor cells on day 8, corresponding to the parasitemia peak.

### Immune mediated specific reversion of the antitumor effects of an epimastigote extract

Epimastigotes are *T. cruzi* non-infective forms, obtained from axenic cultures [30]. They express nTcCRT, although their capacity to translocate the chaperone to the area of flagellum emergence is absent or marginal [31]. We first asked whether an epimastigote extract (EE) reproduces the in vivo antitumor effect of infective trypomastigotes and if this effect is inhibited by whole anti-rTcCRT Abs, based on the rationale described at the beginning of this section. The Tulahuén clone (donated by Dr. Juan Diego Maya, ICBM, Faculty of Medicine, University of Chile), was grown at 28 °C in modified Diamond’s medium [30] and 2×10^6^ parasites were used to prepare the EE, by standard procedures [32].

Four experimental groups were designed. All of them were inoculated with the TA3-MTXR tumor cells. In addition, the animals were treated as follows: Group I: PBS, as positive control (s.c.); Group 2: EE, s.c.; Group 3: In order to reverse the antitumor capacity of EE, this group was inoculated with whole anti-rTcCRT Abs (200 μg); Group 4: As a negative control these mice received preimmune Abs.

All inoculations were performed every other day on a peritumor area.

### Immune mediated specific reversion of the antitumor effects of trypomastigote infection or epimastigote extract, as assessed by histopathology

Tumors were extracted and fixed with 10 % formalin for 48 h, dehydrated and clarified [11]. The sections were embedded on paraffin (Paraplast Plus®, Sigma-Aldrich, USA) and 4 μm thick slices were stained with Hematoxylin-Eosin, and mounted with Entellán®. A description of the main lesions observed in the tumor samples was performed on 10 fields of 40 and 400× zoom, in an Olympus FS100 microscope.

### Statement of bioethical approval

Our Institutional Bioethics Committee approved the experimental protocols using animals. Eight to 12-week-old female A/J mice were maintained under internationally accepted guidelines in our Animal Facility (Faculty of Medicine, University of Chile).

### Statistical analyses

When pertinent, experimental data were statistically validated by One or Two-Way ANOVA and *post-hoc* Bonferroni’s Multiple Comparison and also by one-tailed Wilcoxon Signed-Rank, using the software GraphPad Prism 5.

## Results

### rTcCRT and its N-domain interactions with ECs can be interfered with Abs or Fucoidan

**Fig. 1:**
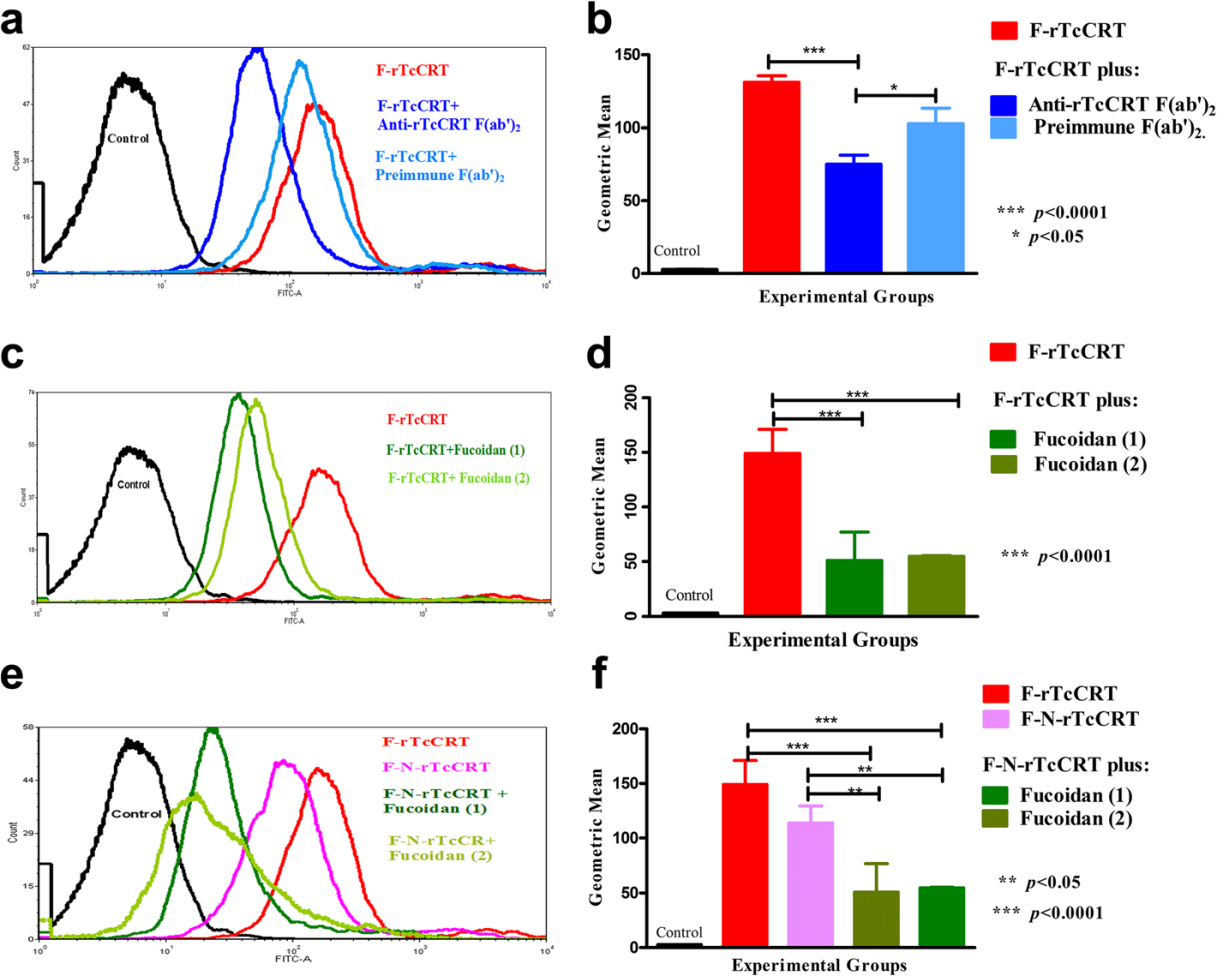
The interaction of rTcCRT and its N-domain with ECs can be intervened by Abs or Fucoidan. Using flow cytometry, human ECs (EAhy926) were incubated with F-rTcCRT in the presence of: **a** Anti-rTcCRT F(ab’)_2_ fragments or their preimmune counterparts; **c** Fucoidans 1 or 2. EAhy926 cells were also incubated with: **e** F-N-rTcCRT or alternatively with F-N-rTcCRT and Fucoidans 1 or 2. Statistical validation: **b d** and **f** (One-Way ANOVA and a *post-hoc* Bonferroni’s multiple comparison test

rTcCRT contact with ECs is a prerequisite for the inhibition of cell proliferation and migration, two crucial events in angiogenesis [9]. Most likely rTcCRT interacts with SRs present on human ECs [21–23]. In such a case, Abs against rTcCRT or, fluid-phase Fucoidans (specific SR ligands), should interfere with the EC/rTcCRT interaction in vitro. Figure 1a, c show typical flow cytometry analyses, while Fig. 1b, d, summarize their respective statistical validations. Figure 1a shows that anti-rTcCRT F(ab’)_2_ fragments inhibit the F-rTcCRT/EC interaction (*p* < 10^−4^). Figure 1c shows that two types of Fucoidans do interfere with this interaction (*p* < 10^−4^), as valid positive controls. The F-N-rTcCRT interaction with ECs was similarly inhibited by Fucoidan 1 and Fucoidan 2 (*p* < 0.05, in both cases). Moreover, and as expected, F-rTcCRT and F-N-rTcCRT interactions with ECs were similar (Fig. 1e-f).

### Whole anti-rTcCRT Abs and their F(ab’)_2_ fragments abrogate the rTcCRT-dependent inhibition of EC proliferation

**Fig. 2:**
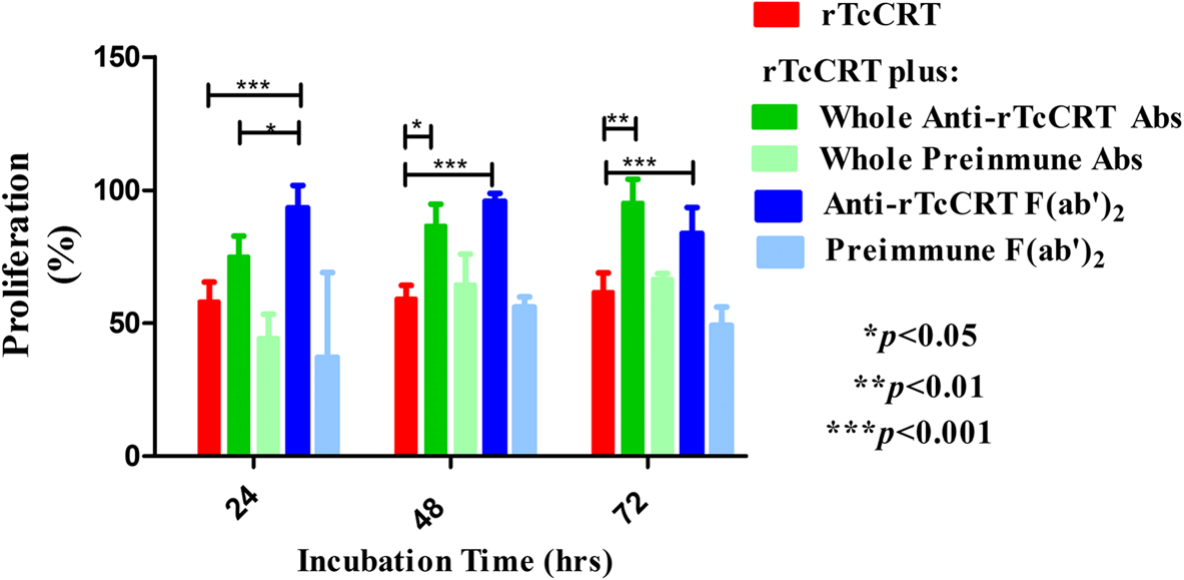
Whole anti-rTcCRT Abs and their F(ab’)_2_ fragments revert the rTcCRT-dependent inhibition of EC proliferation. Human ECs (EAhy926) were incubated with rTcCRT, in addition to whole anti-rTcCRT Abs, their respective F(ab’)_2_ fragments or the preimmune counterpart of each Ab type. Proliferation was analyzed by incorporation of BrdU and expressed as percentage, where 100 % corresponds to cells incubated with medium alone (Two-Way ANOVA and a *post-hoc* Bonferroni’s multiple comparison test)

Since, as shown above, rTcCRT interaction with ECs is reversed by anti-rTcCRT Abs (and also by fluid-phase Fucoidans), we asked whether these Abs also abrogate the chaperone capacity to inhibit EC proliferation. We determined that, while the F(ab’)_2_ Ab fragments inhibited ECs proliferation at 24, 48 and 72 h of incubation (*p* < 0.001, in all cases), the inhibitory capacity of their whole counterparts was detectable only at 48 and 72 h (*p* < 0.05 and *p* < 0.01, respectively) (Fig. 2). Since these experiments were conducted in the absence of C1, it is then possible that these Abs interfere with the rTcCRT capacity to interact with SRs present on ECs.

### Immune mediated specific abrogation of the anti mammary tumor effects of rTcCRT

**Fig. 3:**
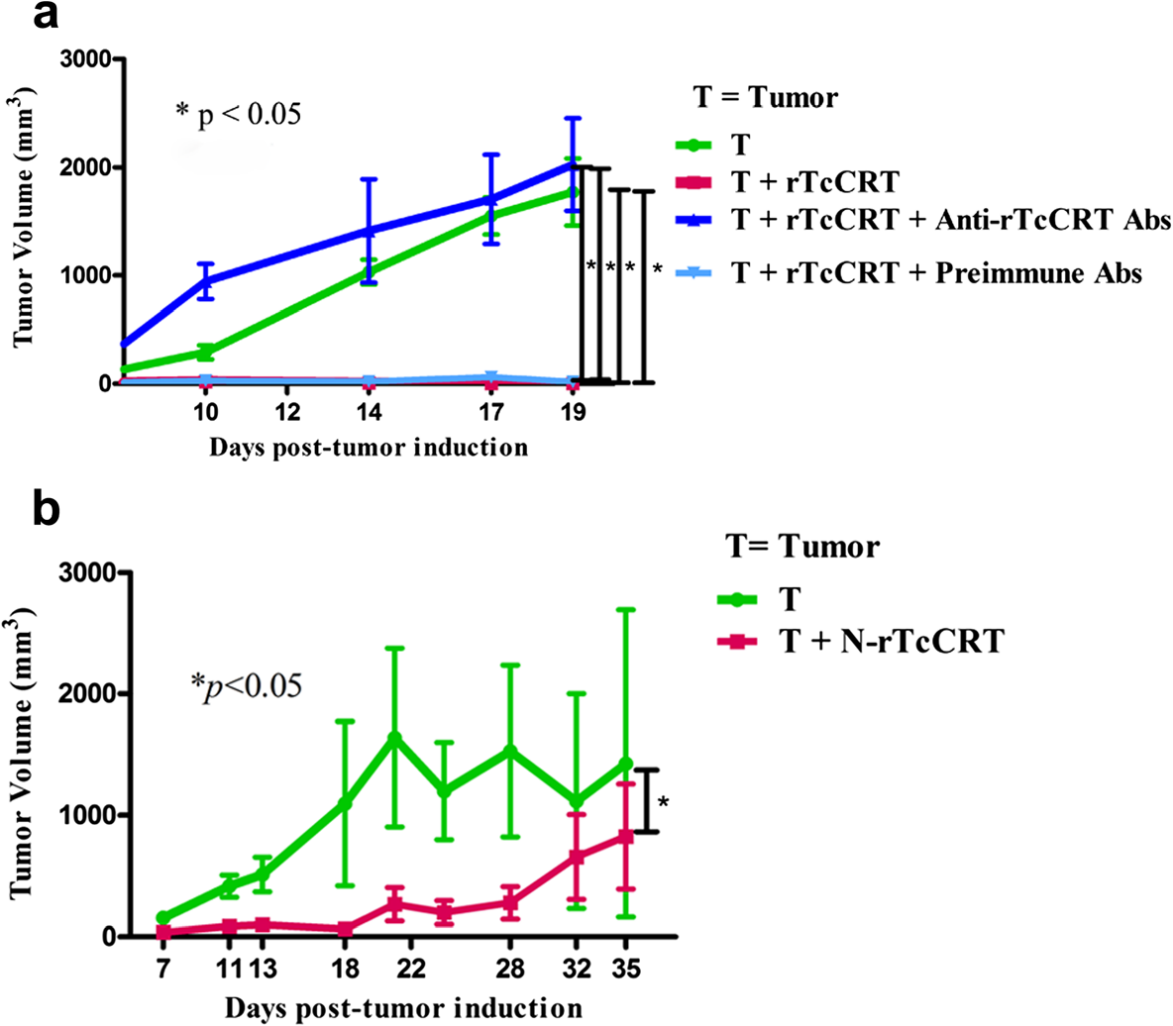
The anti mammary tumor effect of rTcCRT is reversed by whole anti-rTcCRT Abs and the N-rTcCRT domain retains the effect of the full recombinant protein. A/J mice were inoculated s.c., in a supra escapular area, with mammary adenocarcinoma cells (TA3-MTXR, control group) and challenged as indicated in the Figures and detailed in the Materials and Methods section. **a** Reversion of the rTcCRT antitumor effect; **b** N-rTcCRT antitumor effect. Recombinant proteins and Abs were delivered every other day. (One-tailed Wilcoxon signed-rank test)

If the in vivo antitumor effect of *T. cruzi* infection is indeed mediated by translocated/exteriorized nTcCRT [16], this effect should be reversed by anti-rTcCRT Abs. We first validated these Abs with regard to their capacity to revert the antitumor effect of rTcCRT, already reported by our laboratory [[9–12]. Indeed, this property was abrogated by these Abs, and tumor growth reached levels similar to those of the negative control group (Fig. 3a). (Noteworthy, this experiment also confirms the potent antitumor effect reported for rTcCRT [9, 11], used here as a positive control). Additionally and as expected, the N-rTcCRT domain maintains the antitumor effect of its whole counterpart, as shown in Fig. 3b.

### Specific anti-rTcCRT Abs abrogate the mammary antitumor effects of *T. cruzi* infection

**Fig. 4:**
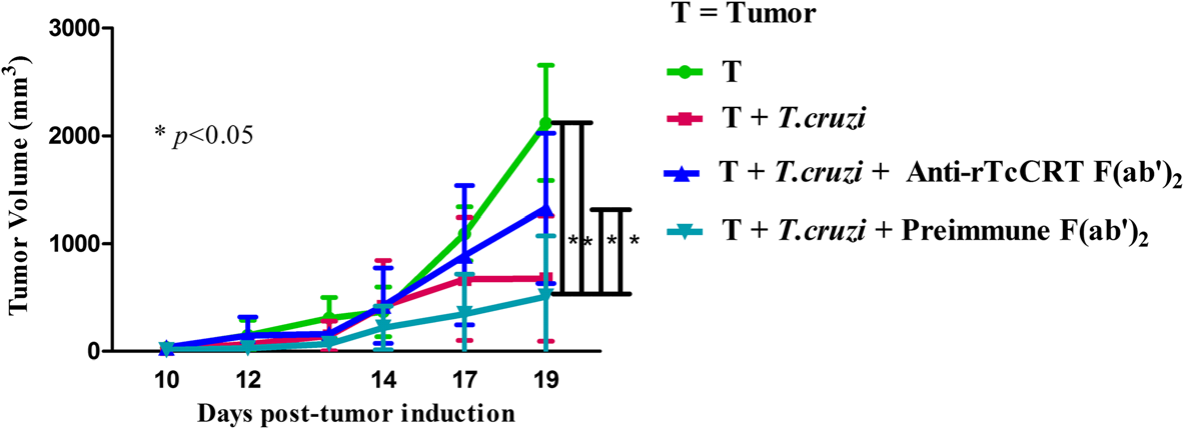
The anti mammary tumor effects of *T. cruzi* infection is reversed by specific anti-rTcCRT F(ab’)_2_ fragments. A/J mice were inoculated s.c. in a supra escapular area with mammary adenocarcinoma cells (TA3-MTXR, control group) and challenged as indicated in the Figure and detailed in the Materials and Methods section. In all cases the infection with *T. cruzi* was performed 8 days before the inoculation of tumor cells, and the delivery of Abs was performed every other day. (One-tailed Wilcoxon signed-rank test)

The rationale for choosing F(ab’)_2_ Ab fragments to revert the antitumor effects of a trypomastigote infection is based mainly on the fact that *T. cruzi* translocates its TcCRT to the area of parasite flagellum emergence, where it binds complement C1 [15, 33]. The Fc-dependent IgG capacity of whole anti-rTcCRT Abs to recruit additional C1 is absent in F(ab’)_2_ fragments; thus these fragments block infectivity [25, 33] and possibly the parasite antitumor properties. We therefore propose that this *T. cruzi* translocated nTcCRT contacts ECs, thus exerting an antiangiogenic effect, followed by inhibition of tumor growth. In fact, those animals inoculated with anti-rTcCRT F(ab’)_2_ fragments showed an inhibition of the antitumor effect associated with this infection (Fig. 4). We have thus established a causal relationship between parasite nTcCRT and the antitumor effect of this infection.

### An epimastigote extract displays an anti mammary tumor effect that can be also reversed by specific anti-rTcCRT Abs

The original experiments of Roskin and Klyuyeva included the direct inoculation, at the tumor site, of a lysate of blood trypomastigotes, with antitumor consequences (reviewed in [2]). We used EE (prepared with non-infective parasites) instead, that displayed a single band, compatible with nTcCRT, when detected with specific polyclonal and monoclonal Abs, in conventional immunowesternblotting [34]. We inoculated the animals with whole anti-rTcCRT Abs, aiming at neutralizing the EE parasite protein capacity to inhibit tumor growth.

**Fig. 5:**
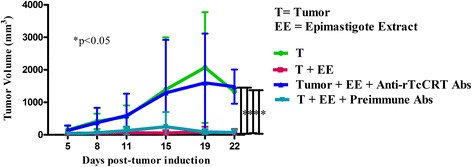
The anti mammary tumor effect of an epimastigote extract (EE) is reversed by specific anti-rTcCRT Abs. A/J mice were inoculated s.c. in a supra scapular area with mammary adenocarcinoma cells (TA3-MTXR, control group) and challenged as indicated in the Figure and detailed in Materials and Methods. The EE anti mammary tumor effect is reverted by specific anti-TcCRT Abs. (One-tailed Wilcoxon signed-rank test)

As predicted, in the EE-treated group, smaller tumor sizes and even full tumor regression was observed. When anti-rTcCRT Abs were inoculated, this EE effect was statistically reversed (Fig. 5).

### Mammary tumors grown in animals alternatively treated with *T. cruzi* infection, EE or N-rTcCRT, display a less aggressive histological pattern

**Fig. 6:**
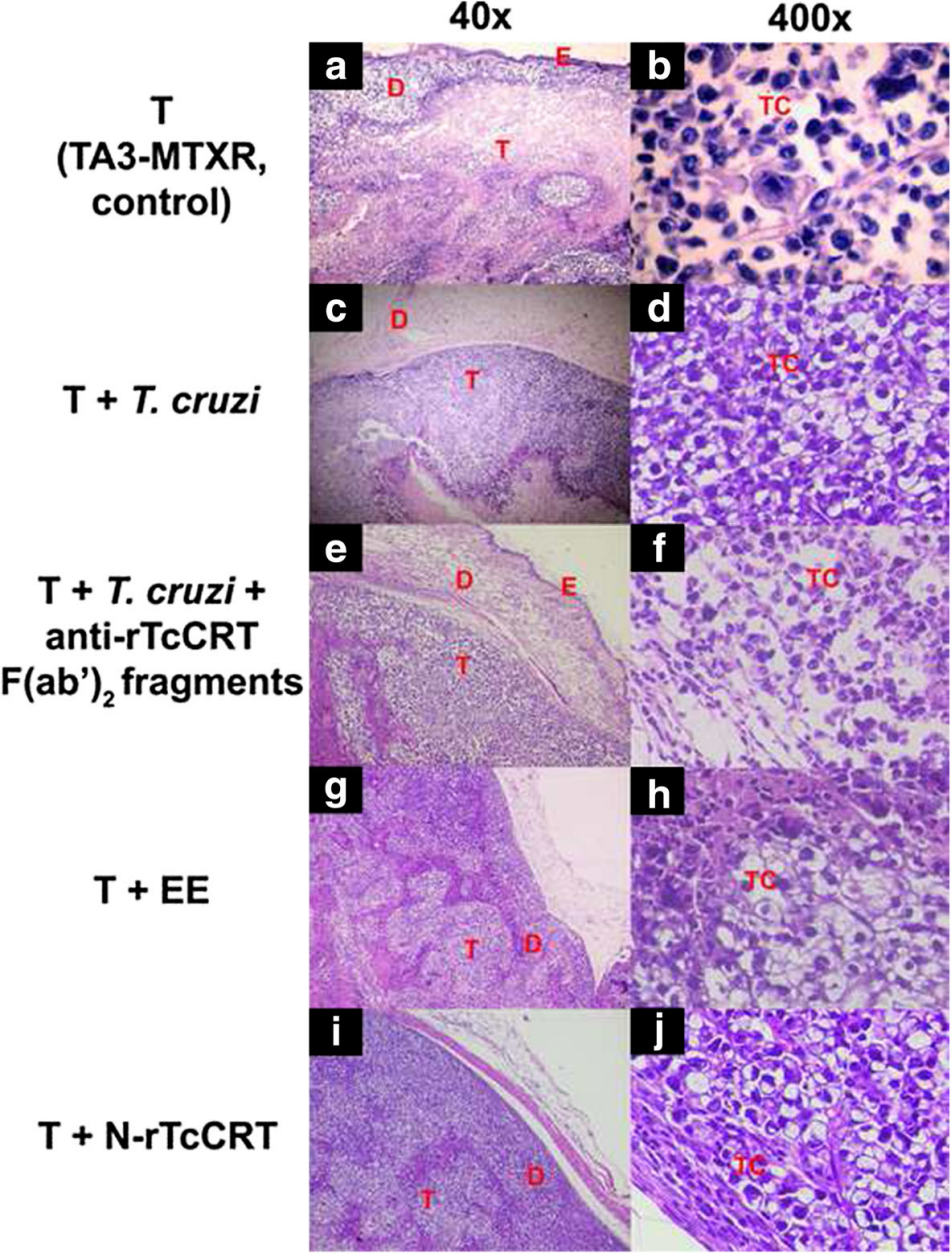
Mammary tumors from animals treated with either *T. cruzi* infection, EE or N-rTcCRT, display less aggressive histological patterns. TA3-MTXR tumor (T) extracted from representative animals: **a b** Non infected, control group; **c d** *T. cruzi* infected; **e f** *T. cruzi*- infected, inoculated with anti-rTcCRT F(ab’)_2_ fragments; **g h** Inoculated with an EE; **i j** Inoculated with N-rTcCRT. The following parameters were analyzed: E: Epidermis; D: Dermis layer; T: Tumor tissue (**a**, **c**, **e**, **g**, and **i**) and TC: Tumor cells (**b**, **d**, **f**, **h** and **j**)

Mammary tumors, in untreated groups, showed more aggressive histological patterns, with changes at the deep dermal levels (vascularization, edema, inflammation and congestion), with areas of high tumor cell proliferation that profusely invaded the dermal layers (Fig. 6a). Notably, in these untreated animals, tumor cells displayed a less cohesive pattern, with infrequent contacts among them and with a high nucleus/cytoplasm ratio (Fig. 6b). Moreover, a high percentage of bizarre, both multinucleated or mitotic cells, was observed, as well as tumor cells that invaded the dermis and epidermis causing, in some cases, ulceration of the skin surface (Additional file 1: Fig. S1A-D).

On *T. cruzi* infected animals, we found more conserved and differentiated tumor cells, distributed as large sheets of cells in a more compact arrangement, with bigger and vacuolated cytoplasm (Fig. 6d). These samples were less invasive towards adipose tissue and most samples presented defined and encapsulated internal margins, without subcutaneous invasion (Fig. 6c). A similar situation was observed in animals inoculated with EE and N-rTcCRT (Fig. 6g–j). However, when the animals were treated with anti-rTcCRT F(ab’)_2_ fragments, this pattern was not maintained and tumor histopathology was very similar to the control group (Fig. 6e–f).

## Discussion

We have proposed that rTcCRT [13, 14] provides an important at least partial explanation of why *T. cruzi* experimental infection or the inoculation of parasite extracts exerts toxic effects on different tumor types, mammary adenocarcinoma among them (reviewed in [2]).

We have described a 45 kDa *T. cruzi*, highly pleiotropic chaperone protein, TcCRT [13]. rTcCRT [13] is antiangiogenic because it inhibits ECs proliferation, migration and morphogenesis, in several in vitro, *ex vivo* and in vivo assays, in 3 species, *H. s. sapiens* included [9, 16, 17]. Furthermore, in vivo, rTcCRT inhibits the growth of an experimental mammary adenocarcinoma, when inoculated in an area near the tumor. This effect is more potent than the one elicited by its rHuCRT counterpart [9]. However, the proposal that *T.cruzi* infection has an antitumor effect mediated by its nTcCRT, needs a formal demonstration, as justified in the Introduction section. This important question was addressed herein. In order to causally implicate nTcCRT in the antitumor effect of *T. cruzi* infection, we blocked these effects, with polyclonal anti-rTcCRT Abs, in the context of the trypomastigote infection or epimastigote extract (EE) inoculation. Since angiogenesis is important for tumor development, we previously showed that these Abs neutralize the antiangiogenic effect of rTcCRT [17].

We first checked whether anti-rTcCRT Abs block the chaperone binding to ECs [9], since this contact is a necessary prerequisite for angiogenesis inhibition. As expected, anti-rTcCRT F(ab’)_2_ fragments reversed this interaction (Fig. 1a-b), as well as the antiproliferative effect reported for rTcCRT [9] (Fig. 2). Thus, we validated these Abs for their use in vivo.

The rationale for choosing whole anti-rTcCRT Abs or their F(ab’)_2_ fragments, in the experiments described below, is based on their differential Fc-dependent capacity to bind human complement C1 [25, 33] Thus: i). Whole anti-rTcCRT Abs, upon recognizing translocated nTcCRT on trypomastigotes, will recruit host C1, a potent infectivity-promoter [33]; ii). Because this Fc-dependent C1-recruiting IgG capacity is absent in F(ab’)_2_ fragments, these will block infectivity [33]; iii). Contact of rTcCRT with C1 and/or membrane receptors, and its functional consequences, should be blocked with both whole IgG or F(ab’)_2_ fragments [33] and, iv). nTcCRT, present in a non-infective EE [34] should be also blocked by both whole IgG or F(ab’)_2_ fragments (Figs. 5 and 4, respectively).

A possible mechanism favoring the rTcCRT EC internalization [9] involves its binding to Scavenger Receptors (SRs). nTcCRT has affinity for collagenous structures, thus explaining its binding to human C1, with consequent inhibition of the classical pathway of the complement system [15, 33]. Fluid-phase Fucoidan, bearing extensive collagen-like sequences, inhibits the binding of CRT to SR-A, present on phagocytic cells [18], and the internalization of rTcCRT by ECs [9]. In agreement with these findings, the two types of Fucoidans used by us, inhibited the binding to ECs of both rTcCRT (Fig. 1c-d) and its N-domain (Fig. 1e-f).

Since SR-A1 has a high proportion of collagenous sequences [22, 23], the possibility exists that rTcCRT exerts its action via EC internalization through this SR [21–23]. The relevant signaling pathways and activated or inhibited genes are still unknown.

Since anti-rTcCRT Abs abrogated the antitumor effect of rTcCRT [9–11] (Fig. 3a), it could be proposed that they should block the native parasite protein, in the context of an infection or EE inoculation.

We next asked whether antiangiogenesis has implications in the antitumor capacity of the parasite chaperone. We showed that the N-domain (antiangiogenic) [9] effectively inhibited tumor development (Fig. 3b), thus opening the probability that even smaller subdomains, still to be defined, may also exert this effect.

However, the facts that, on the one hand, *T. cruzi* infection has an antitumor effect [2–5] and, on the other, rTcCRT largely reproduces it [9], does not necessarily mean that, during infection, nTcCRT is indeed a valid mediator of this effect. As expected, anti-rTcCRT (Fab’)_2_ Abs inhibited this antitumor effect (Fig. 4), thus formally involving nTcCRT.

Roskin and Klyuyeva injected, directly into the tumor site, parasite lysates with antitumor effects (reviewed in [2]). Epimastigotes, non-infective *T. cruzi* forms, express marginal levels of TcCRT on their external membrane [31], although intracellular levels of the chaperone are apparently normal. TcCRT translocation in these parasite forms is apparently deficient [31] and may explain, at least partly, their incapacity to infect cells. As expected, EE delayed tumor growth and, in some animals, complete tumor regression was observed (Fig. 5). A recent report [6] shows similar results, in a rat model. Indeed, they reproduce the antitumor effects of *T. cruzi* epimastigote lysates and found that they trigger cytotoxic responses against tumors, with activation of both CD4^+^ and CD8^+^ T cells. These extracts elicited the production of antibodies that cross reacted with human colon and breast cancer tissue samples. Given our results, most likely the antitumor effect observed by these authors should be significantly and specifically inhibited by our anti-rTcCRT Abs, as shown in Fig. 5. In synthesis and in agreement with the previous results, *T. cruzi* infection effects on the normal course in a mammary tumor development is largely nTcCRT-dependent.

Representative tumor histology indicated decreased tumor malignancy, in particular at 28 d.p.i. Noteworthy, in *T. cruzi* infected animals, or EE or N-TcCRT inoculated ones, tumors were encapsulated and less invasive (Fig. 6c-d, g–j). In agreement with the previous results, when, in infected animals, TcCRT was blocked with F(ab’)_2_ anti- TcCRT Ab fragments, more aggressive tumor patterns are observed (similar to the control group) (Fig. 6a-b, as compared to Fig. 6e-f).

Altogether, these results support the antitumor activity of nTcCRT, although we cannot completely rule out the involvement of other parasite molecules.

The use of a recombinant nonpathogenic *T. cruzi* clone as a vaccine vector to induce T-cell-mediated immunity has been reported [35]. These authors show that transgenic parasites, expressing a cancer testis antigen, elicit human antigen-specific T-cell responses in vitro and protection against a murine melanoma in vivo. Given our results, in this work it would have been important to define whether the nonpathogenic *T. cruzi* clone used by these authors translocates-externalizes its CRT. As mentioned above, non-infective epimastigotes are strongly impaired in their capacity to translocate this chaperone [31]. Moreover, hemiallelic *TcCRT* KO, wild type and transgenic parasites respectively carrying one, two and three *TcCRT* gene copies, express increasing levels of the protein. As expected, these facts positively correlate with increased in vitro resistance to human complement, as well as with infectivity [36, 37]. We will soon test these three clones in their experimental capacity to control tumor growth.

Although we cannot rule out a role of antitumor innate immunity in the results presented herein, during infection TcCRT seems to be playing a direct immunogenic role in the induction of specific anamnestic immunity against the experimental mammary tumor tested. Human complement component C1 plays an extremely important adjuvant role by generating potent opsonizing signals on the parasite [33, 38], thus increasing their infectivity. Preliminary results from our laboratory, show increased numbers of CD8^+^ and CD4^+^ lymphocytes in rTcCRT-treated mammary tumor bearing mice. Again, concomitant important decreases in tumor sizes were observed (Gallardo et al., 2016, unpublished).

**Fig. 7:**
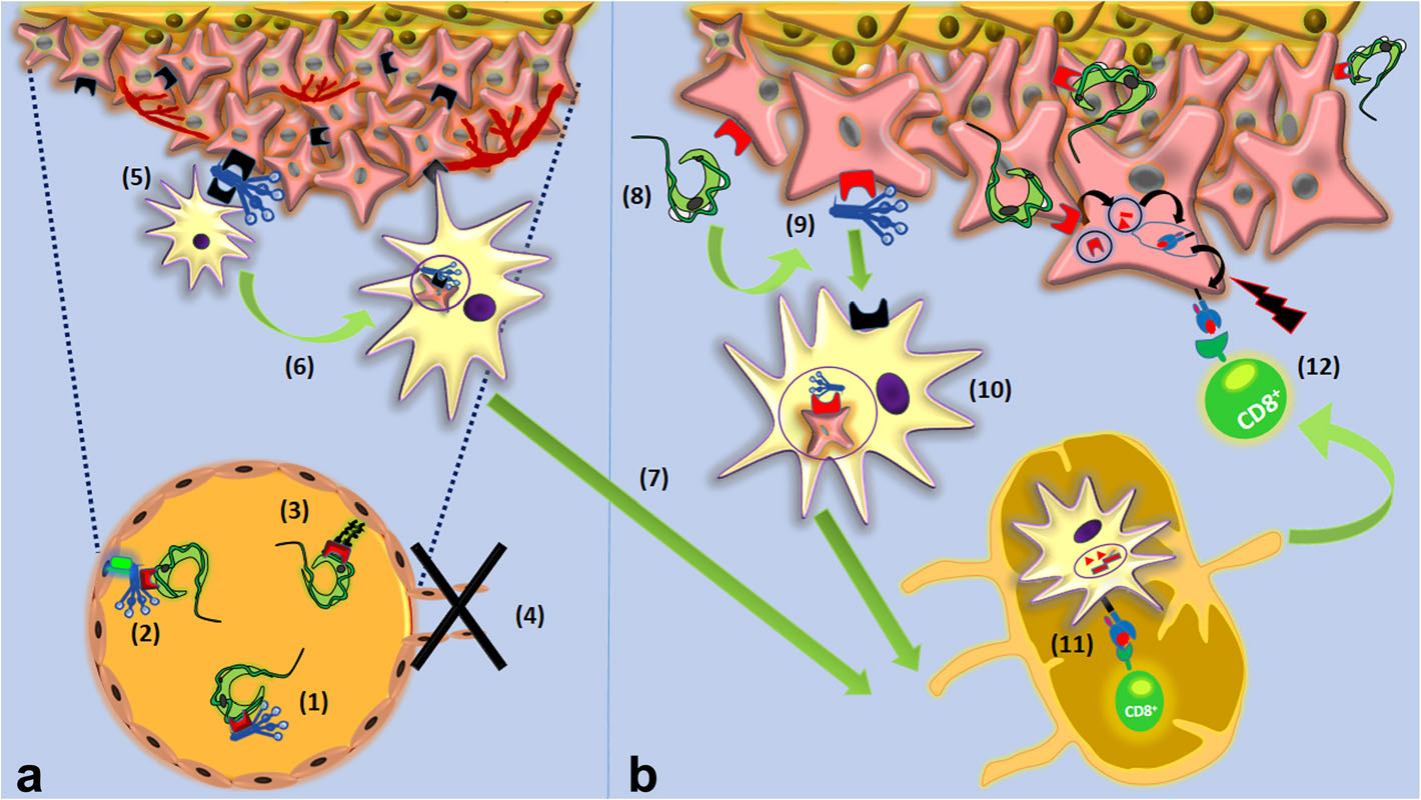
Proposed extracellular mechanisms explaining, at least partially, the in vivo antitumor effect of *T. cruzi* infection. **a** TcCRT antiangiogenic effect: (1) *T. cruzi* exposes its CRT on the parasite surface, followed by C1 recruitment and inactivation; (2) On the ECs membrane, a trimolecular synapse is formed by cC1qR-C1-TcCRT, from EC, host and parasite origins, respectively; (3) SRs present on ECs bind TcCRT. The last two mechanisms, could activate antiangiogenic signals on ECs, with consequent inhibition of angiogenesis (4). Decreased nutrients and oxygen supply may constitute a stressful environment for the tumor, CRT will be exteriorized and C1 recruited (5). Dendritic antigen presenting cells (APCs), through their cC1qRs will recognize, internalize and process these tumor cells (6) on their way to local lymph nodes (7). **b** Possible nTcCRT-mediated induction of an anamnestic antitumor immune response: Parasite translocated TcCRT binds to the tumor cell (8), with subsequent capture of host C1 (9). This C1 will be recognized by cC1qR present on APCs, followed by internalization of this complex (10). Among many other possibilities, APCs will cross-process TcCRT and specific peptides from this parasite protein will be loaded onto MHC I molecules. APCs will enter the regional lymph node and present these nTcCRT-specific peptides to cytotoxic T lymphocytes, thus leading to their activation (11). These CD8^+^ cytotoxic T lymphocytes will leave the lymph node and kill tumor cells that also present TcCRT derived peptides (12)

In synthesis, as depicted in Fig. 7, we can propose that *T. cruzi* infection mediates key alterations in the tumor cell microenvironment that may lead to an adaptive immune response, with significant antitumor effects. Once in the circulation, *T. cruzi* must swiftly invade ECs. Translocated-exteriorized TcCRT [15] will recruit and inactivate plasma complement C1. This will allow the parasite to contact ECs via cC1qR [19, 20]. Otherwise, the chaperone protein could interact directly with SR-A1 on ECs [21–23]. Both pathways may lead to antiangiogenesis and generate a stressful environment where tumor cells will externalize their CRT, as previously shown with other stressing agents, such as antracyclins [39]. Complement C1 recruitment and increased tumor cell phagocytosis by dendritic cells will follow.

On the other hand, an adaptive immune response may involve both highly immunogenic rTcCRT, and its native counterpart, this one present on the membrane of infecting trypomastigotes [15] or in EE [32, 34]. In any case, the chaperone protein should reach the surface of both endotheliocytes and tumor cells, thus generating a site for C1 binding. Tumor cells, thus opsonized should be phagocytized by dendritic cells. Upon arrival to the regional lymph nodes, these dendritic cells will present antigenic peptides derived from TcCRT, thus activating cytotoxic T lymphocytes. Whether tumor cells can cross-present peptides derived from endocytosed TcCRT to cytotoxic T cells, is a matter of current research in our laboratory. Activated cytotoxic T cells should then return to the tumor site and act against neoplastic tumor cells in both, primary, as well as in metastatic foci (Fig. 7), a mechanism already shown for whole EE [40].

Because about 90 % identity exists among mammal CRTs, while TcCRT is only 50 % identical, the latter is highly immunogenic in infected humans [34] and in animals [41]. Interestingly, TcCRT sequence is closer to plant CRT (e.g. *Arabidopsis thaliana*) than to mammal CRT (Weinberger et al., 2016, unpublished), thus explaining its high immunogenicity. The parasite protein (recombinant or native) bound to mammal tumor cells, could thus provide strong immunogenic signals that reveal the tumor presence to an otherwise tolerant immune system.

In a large set of experimental animals treated with rTcCRT, no clinical deleterious effects have been detected by standard clinical veterinary criteria. rTcCRT, or derived domains, are interesting immunological tools to be considered in more advanced preclinical trials (e.g. rTcCRT and C1 binding to human mammary tumor cell lines and its opsonizing consequences).

## Conclusions

Translocated/externalized native *Trypanosoma cruzi* calreticulin is responsible for at least an important part of the anti mammary tumor effect of the experimental infection with this parasite. Highly immunogenic recombinant TcCRT largely reproduces this effect mainly by capturing complement C1 on the tumor cell surface, where a danger opsonizing signal is thus generated. Altogether, these results allow us to propose that an extremely relevant alteration in the tumor microenvironment is thus accomplished. The presence of the tumor cells is thus revealed to the immune system, with likely generation of an anamnestic systemic immune response. These results identify recombinant TcCRT or domains thereof, as interesting tools for further preclinical studies in mammary cancer (i.e. tissue specificity of the parasite chaperone, signal transduction pathways involved, rTcCRT opsonization of human tumor cell lines).

## Additional file

Additional file 1: Fig. S1. Quantitative analysis of necrotic area (A), mitosis index (B) ulceration (C) and bizarre cells (D) in slides of tumors from different groups stained with hematoxylin and eosin. The percentages (A, C) or absolute numbers (B, D) were calculated in triplicates in 10 fields with a 400× magnification. (One-Way ANOVA and a *post*-*hoc* Bonferroni’s multiple comparison test). (TIF 2723 kb)

## Abbreviations

Abs: Antibodies
BrdU: Bromodeoxyuridine
cC1qR: C1 receptor
CRT: Calreticulin
EC: Endothelial cell
EE: Epimastigote extract
ER: Endoplasmic reticulum
FBS: Fetal bovine serum
F-rTcCRT: FITC-coupled recombinant *trypanosoma cruzi* calreticulin
HuCRT: Human calreticulin
MHC I: Major histocompatibility complex class I
MmCRT: Murine calreticulin
N-rTcCRT: Recombinant N-domain of *trypanosoma cruzi* calreticulin
nTcCRT: Native *trypanosoma cruzi* calreticulin
rTcCRT: Recombinant *trypanosoma cruzi* calreticulin
SR: Scavenger receptor
*T. cruzi*: *Trypanosoma cruzi*
TA3-MTXR: Methotrexate multiresistant mammary adenocarcinoma cell line
TcCRT: *Trypanosoma cruzi* calreticulin
