# Supplementary figures and images for: Does native Trypanosoma cruzi calreticulin mediate growth inhibition of a mammary tumor during infection?
Source: BMC Cancer. 2016 Sep 13;16(1):731. doi: 10.1186/s12885-016-2764-5 (PMC5020520; doi:10.1186/s12885-016-2764-5)

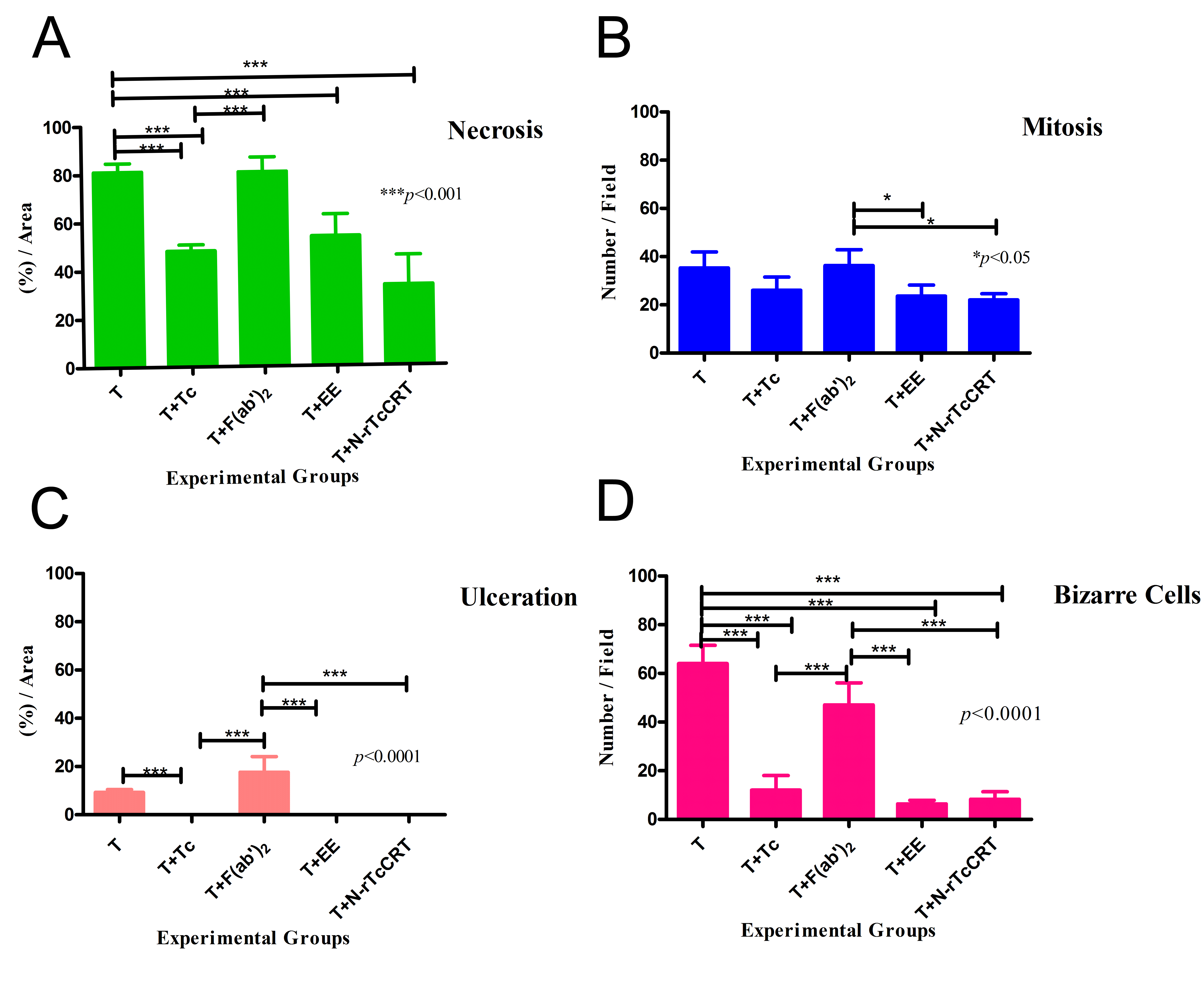

Supplement: Additional file 1: Fig. S1. — Quantitative analysis of necrotic area (A), mitosis index (B) ulceration (C) and bizarre cells (D) in slides of tumors from different groups stained with hematoxylin and eosin. The percentages (A, C) or absolute numbers (B, D) were calculated in triplicates in 10 fields with a 400× magnification. (One-Way ANOVA and a post-hoc Bonferroni’s multiple comparison test). (TIF 2723 kb) [file 12885_2016_2764_MOESM1_ESM.tif]
